# Hyperbaric Oxygen Treatment Ameliorates Hearing Loss and Auditory Cortex Injury in Noise Exposed Mice by Repressing Local Ceramide Accumulation

**DOI:** 10.3390/ijms20194675

**Published:** 2019-09-20

**Authors:** Yu-Ting Su, Yi-Bin Guo, Yao-Ping Cheng, Xi Zhang, Xiao-Ping Xie, Yao-Ming Chang, Jun-Xiang Bao

**Affiliations:** Department of Aerospace Hygiene, Fourth Military Medical University, Xi’an 710032, Shaanxi, China; sssusyt@163.com (Y.-T.S.); gyyb036@163.com (Y.-B.G.); cyp19890316@163.com (Y.-P.C.); zhangxi2015@fmmu.edu.cn (X.Z.); xielaoshi133@126.com (X.-P.X.); changzhuren@126.com (Y.-M.C.)

**Keywords:** hyperbaric oxygen therapy, noise, hearing loss, ceramide, acid sphingomyelinase, acid ceramidase, oxidative stress

## Abstract

Noise-induced hearing loss (NIHL) relates closely to auditory cortex (AC) injury, so countermeasures aiming at the AC recovery would be of benefit. In this work, the effect of hyperbaric oxygen treatment on NIHL was elucidated, which was imposed on mice before (HBOP), during (HBOD) or after (HBOA) noise exposure. Morphology of neurons was assayed by hematoxylin-eosin or Nissl staining. Ceramide (Cer) level was measured through immunohistochemistry analysis. Apoptotic neurons were counted using transferase-mediated dUTP nick end labeling (TUNEL) staining. We demonstrated that the intense, broad band noise raised the threshold of auditory brainstem response, evoked neuronal degeneration or apoptosis and triggered the Cer accumulation in AC, all of which were restored significantly by HBOP, but not HBOD or HBOA. Cer over-generation reversed the advantages of HBOP significantly, while its curtailment recapitulated the effect. Next, noise exposure raised the superoxide or malondialdehyde (MDA) production which was blocked by HBOP or Cer repression. Oxidative control not only attenuated the hearing loss or neurodegeneration but, in turn, reduced the Cer formation significantly. In summary, mutual regulation between Cer and oxidative stress underlies the HBOP’s curative effect on hearing loss and neuronal damage in noise-exposed mice.

## 1. Introduction

Noise exposure has relation to complex manifestations such as hearing loss, tinnitus, reduced speech intelligibility and hyperacusis, which is largely ascribed to the neuronal damage in the central auditory pathway [1]. Including us, multiple labs have revealed the massive loss of cell density and apoptosis of neurons in higher auditory structures like medial geniculate body (MGB) and primary auditory cortex (A1) in the wake of intense noise exposure [1,2,3,4,5]. In this respect, keeping the auditory neurons from noise elicited damage stands out crucial for the improvement of the hearing performance.

Hyperbaric oxygen therapy (HBOT), a treatment of breathing 100% oxygen under ambient pressure higher than 1 atmosphere absolute (ata), has become a well-established curative approach for malaises with air embolus or hypoxia present, including decompression sickness, carbon monoxide poison, serious infections, and wounds unhealed owing to diabetes or radiation injury [5]. Recently, HBOT exhibits clear advantages in sicknesses as chronic wound [6,7], diabetic foot [8] and peripheral arterial occlusive disease [9]. Moreover, in addressing the idiopathic sudden sensorineural hearing loss, a context that is accompanied by tinnitus, sensation of fullness, and a blocked ear or vertigo, HBOT proves to be efficient especially being accessed at early stage [10]. Even so, the action of HBOT on noise-induced hearing loss and auditory neuronal damage is still under debate.

Ceramide (Cer) has been regarded as the most pivotal sphingolipid second messenger, governing key cellular processes such as apoptosis, proliferation, differentiation, and motility [11]. Acid sphingomyelinase (ASM) and acid ceramidase (ACDase) are two key enzymes regulating synthesis and degradation of Cer in lysosomes [12]. Our former work has demonstrated the broad-band noise exposure induced Cer accrual in auditory cortex (AC) of mice which underlay the hearing loss as well as neurodegeneration [3]. Among the various downstream events of Cer, oxidative stress acts as the leading one. Cer promotes activation of NADPH oxidase by facilitating aggregation of subunits of gp91-phox and p47-phox [13] and perturbs the mitochondrial complex assembly inducing reactive oxygen species (ROS) production [11]. ROS mediates the apoptotic potency of Cer in a number of cells [14,15,16]. Although HBOT fortifies oxygen tension in multiple tissues, it furthers the accrual of anti-oxidants as well which underlies the mitigation of neuronal damage in a host of maladies [17]. However, whether the Cer governed ROS plays a role in HBOT’s regulation on noise elicited hearing loss or neuronal impairment is unclear.

In the present study, we examined the improvement of auditory sensitivity and neuronal morphology in AC of mice with the HBOT exerted before, during, or after noise exposure. Then, the mechanism pertaining to Cer generation and the oxidative stress was investigated.

## 2. Results

### 2.1. HBOT Corrected Noise-Induced Hearing Loss and Morphologic Disorder in AC of Mice

**Figure 1:**
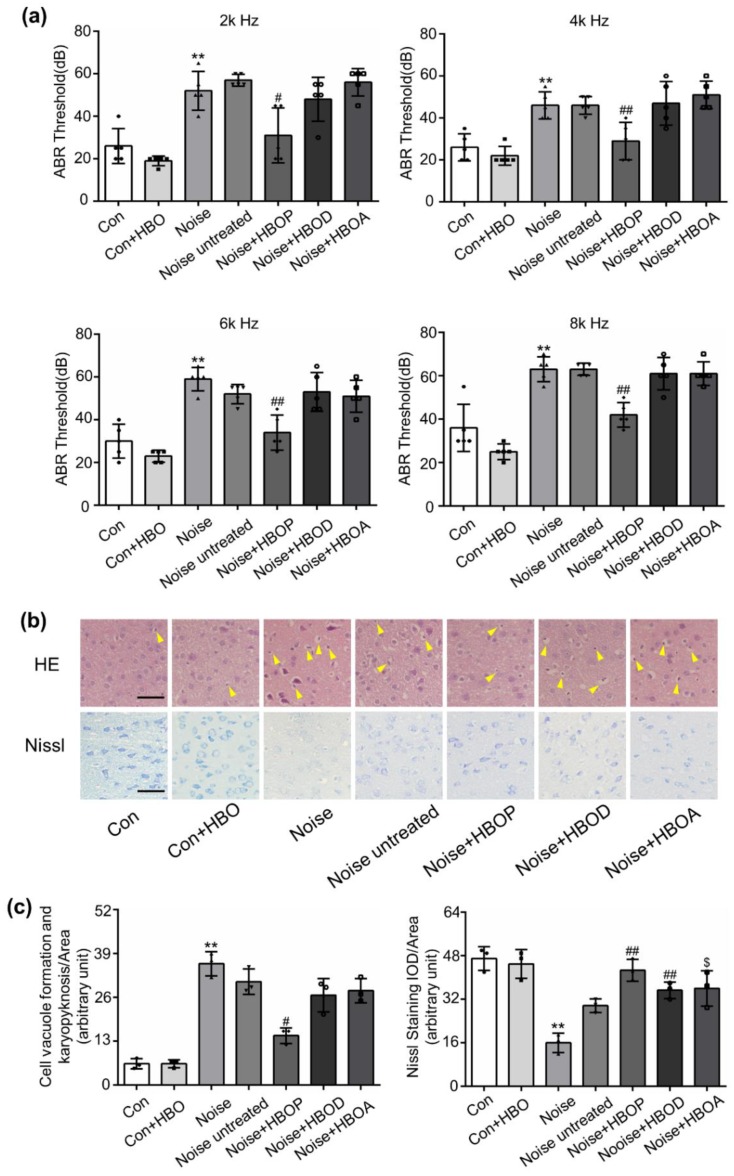
Auditory brainstem response (ABR) threshold to sound with frequency of 2k, 4k, 6k, or 8k Hz (**a**), original images (**b**) and summarized data (**c**) of hematoxylin and eosin (HE) or Nissl staining in auditory cortex (AC) of mice in Con, Con + HBO, Noise, Noise untreated, HBOP, HBOD, and HBOA. The arrows indicate the vacuole formation and karyopyknosis in the neuron, magnification ×400, scale bar = 50 μm. Data is shown as means ± SD, *n* = 5 in ABR, *n* = 3 in HE or Nissl staining, **P < 0.01, *P < 0.05 vs. Con, ^##^*P* < 0.01, ^#^*P* < 0.05 vs. Noise, ^$^*P* < 0.05 vs. Noise untreated. All individual data points were shown in the figure using different shapes, • for Con group, ■ for Con+HBO group, ▲ for Noise group, ▼ for Noise untreated group, ⬥ for Noise+HBOP group, ◯ for Noise+HBOD group, □ for Noise+HBOA group.

To verify the effect of HBOT on hearing loss and neurodegeneration, ABR threshold and neuronal morphology were examined in control and noise-exposed mice. As shown in Figure 1a, after exposure to noise for seven days by 3 h/day, ABR threshold on 2k, 4k, 6k, and 8k Hz was increased, which was restored significantly by HBOP, but not HBOD or HBOA (Figure 1a, *P* < 0.05, *P* < 0.01). With noise stimulation, HE staining detected a substantial morphologic disorder in neurons of AC, including vacuole formation, swollen cell, and nuclei pyknosis, which was similarly reversed by HBOP, but not HBOA or HBOD (Figure 1b,c, *P* < 0.05). Moreover, Nissl staining showed less living neurons in Noise group than Con, which however was reversed by all the three HBOTs (Figure 1b,c, *P* < 0.01, *P* < 0.05).

### 2.2. HBOP Suppressed Neuronal Apoptosis in AC of Noise Exposed Mice

**Figure 2:**
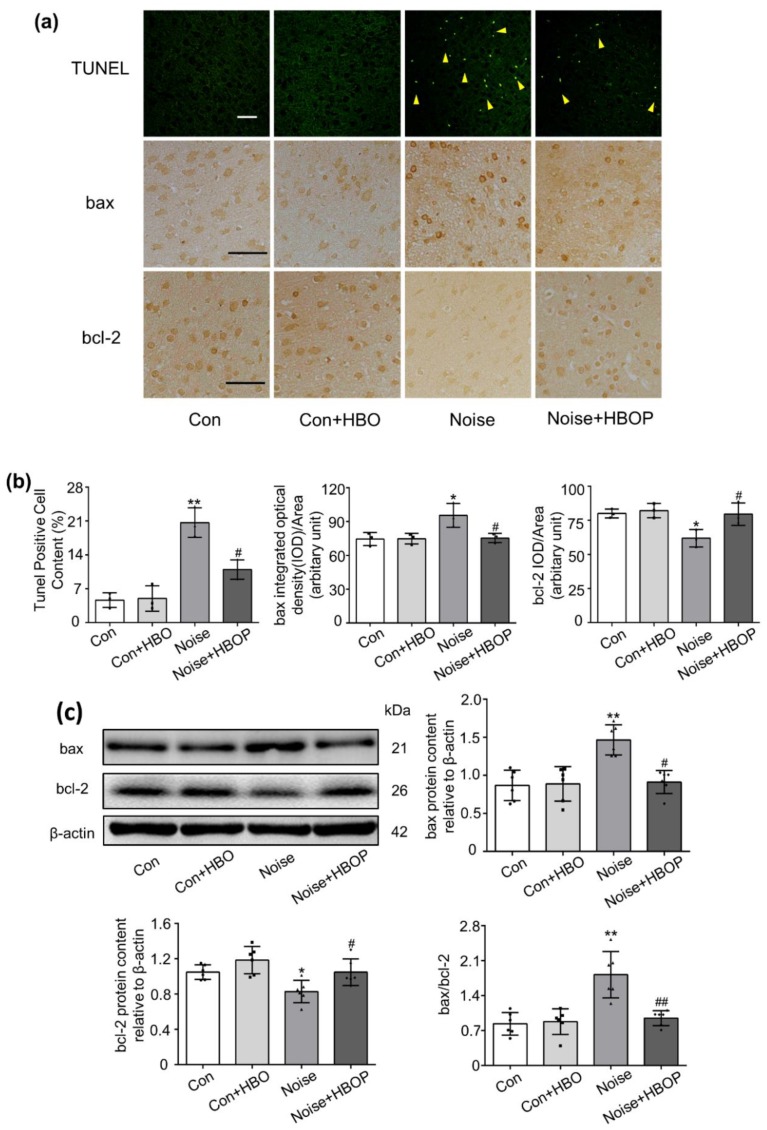
Original images (**a**,**c**) and summarized data (**b**,**c**) showing neuronal apoptosis, protein expression of B cell lymphoma-2 (bcl-2) and bcl-2 associated X protein (bax) in auditory cortex (AC) of mice in Con or Noise group with or without hyperbaric oxygen treatment detected by TdT-mediated dUTP nick-end labeling (TUNEL) (**a**), immunohistochemistry staining (IHC) (**a**) or western blotting (**c**) assay. The arrows indicate apoptotic neurons, in TUNEL, magnification ×200, in IHC, magnification ×400, scale bar = 50 μm. Data are shown as means ± SD, *n* = 3 in IHC or TUNEL, n = 6 in western blot assay, **P < 0.01, *P < 0.05 vs. Con, ##*P* < 0.01, #*P* < 0.05 vs. Noise. All individual data points were shown in the figure using different shapes, • for Con group, ■ for Con+HBO group, ▲ for Noise group, ⬥ for Noise+HBOP group.

As shown in TUNEL test of Figure 2a,b, the proportion of apoptotic neurons was increased by 20.3% after seven days of noise exposure as compared to Con (*P* < 0.01), which was reduced significantly by HBOP (*P* < 0.05). Correspondingly, the protein expression of bax and bcl-2, pro-apoptotic and anti-apoptotic modulators of mitochondrial pathway, were enhanced and reduced, respectively, in noise group relative to Con, both of which were recovered after HBOP was applied (Figure 2a–c, *P* < 0.05, *P* < 0.01).

### 2.3. HBOP Normalized the Cer Generation in AC of Noise Exposed Mice

**Figure 3:**
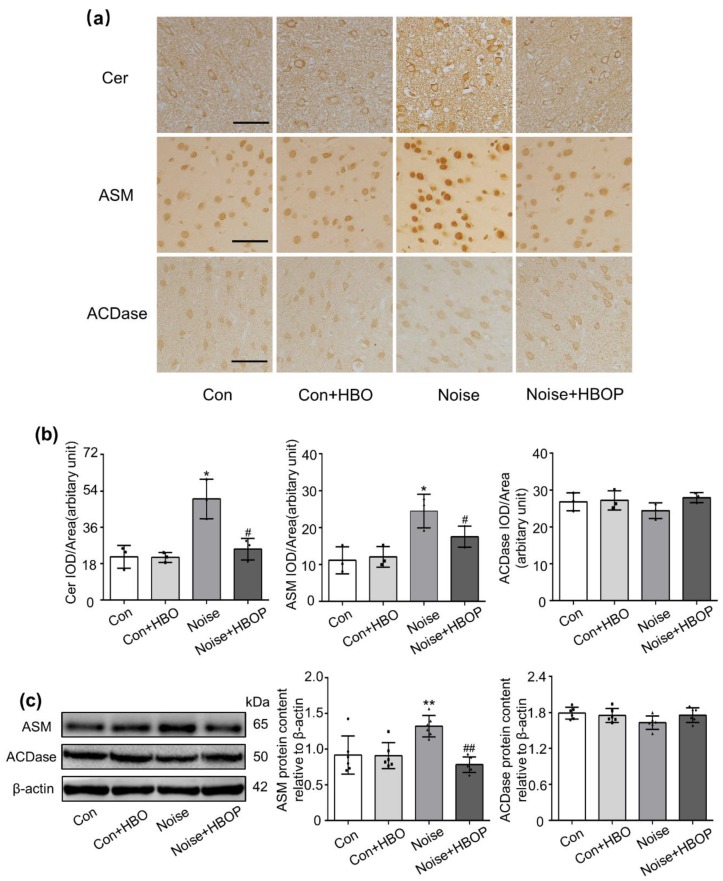
Representative images (**a**) and summarized data (**b**) showing the content of ceramide (Cer), acid sphingomyelinase (ASM) or acid ceramidase (ACDase) in auditory cortex of mice from Con or Noise group with or without hyperbaric oxygen treatment detected by IHC (**a**) and western blot (**c**) assay. Magnification ×400, scale bar = 50 μm. Data are shown as means ± SD, *n* = 3 in IHC, *n* = 6 in western blot, **P < 0.01, *P < 0.05 vs Con, ##*P* < 0.01, #*P* < 0.05 vs Noise. All individual data points were shown. All individual data points were shown in the figure using different shapes, • for Con group, ■ for Con+HBO group, ▲ for Noise group, ⬥ for Noise+HBOP group.

Cer promotes apoptosis in various cells. As shown in Figure 3, Cer abundance was higher in AC of noise-exposed mice than Con (*P* < 0.05), which was recovered significantly by HBOP (*P* < 0.05). Both IHC and western blot analysis indicated that the ASM protein expression was increased significantly (*P* < 0.05) after noise stimulation, which was reduced by HBOP as well (*P* < 0.05). However, HBOT had no influence on ASM expression or Cer content in Con mice. As for the protein expression of ACDase, enzyme responsible for the degradation of Cer, there was no significant difference between Con and Noise groups no matter whether the HBOT was conducted.

### 2.4. Cer Mediated the Curative Effect of HBOP on Noise-Induced Neuronal Damage and Apoptosis

**Figure 4:**
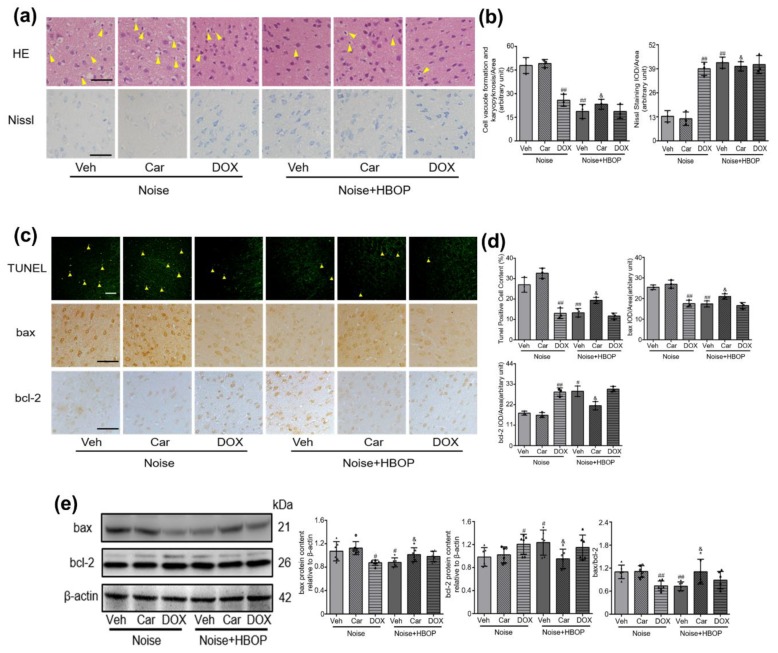
Representative images (**a**) and summarized data (**b**) of HE or Nissl staining in auditory cortex of mice in Noise or HBOP group treated with Veh, Car or DOX. The arrows indicate the vacuole formation and karyopyknosis in the neuron. Original images (**c**,**e**) and analyzed data (**d**,**e**) showing neuronal apoptosis, protein expression of bax and bcl-2 in auditory cortex of mice in Noise or HBOP group treated with Veh, Car, or DOX detected by TUNEL (**c**), IHC (**c**), or western blotting (**e**) assay. The arrows indicate apoptotic neurons, in TUNEL magnification ×200, in IHC, magnification ×400, scale bar = 50μm. Data are shown as means ± SD, n = 3 in HE, Nissl, IHC or TUNEL, n = 6 in western blot assay, ^##^*P* < 0.01, ^#^*P* < 0.05 vs Noise, ^&^*P* < 0.05 vs HBOP. All individual data points were shown in the figure using different shapes, ▲ for Noise group, ◯ for Noise+Car group, ∆ for Noise+DOX group, ⬥ for Noise+HBOP group, ◓ for Noise+HBOP+Car group, ◭ for Noise+HBOP+DOX group.

To explore the role of Cer in HBOP’s curative effect, Doxepin hydrochloride (DOX) and Carmofur (Car) were applied to modify the Cer abundance by inhibiting ASM and ACDase respectively. As shown in Supplementary Figure S4, DOX reduced the Cer in Noise but not HBOP group, while Car increased it in HBOP but not Noise group (*P* < 0.01, *P* < 0.05). Correspondingly, DOX compromised the neuronal disorganization and restored the vitality in AC of Noise group, while Car exacerbates both of them in HBOP group (Figure 4a,b, *P* < 0.01, *P* < 0.05). Figure 4c–e showed that the DOX diminished percentage of apoptotic cell, reduced protein expression of bax and enhanced that of bcl-2 in noise-exposed mice (*P* < 0.01, *P* < 0.05). Conversely, Car augmented apoptotic percentage, increased bax expression and lowered bcl-2 in HBOP group (*P* < 0.01, *P* < 0.05).

### 2.5. HBOP Restrained the Oxidative Stress in AC of Noise Exposed Mice by Regulating Cer

**Figure 5:**
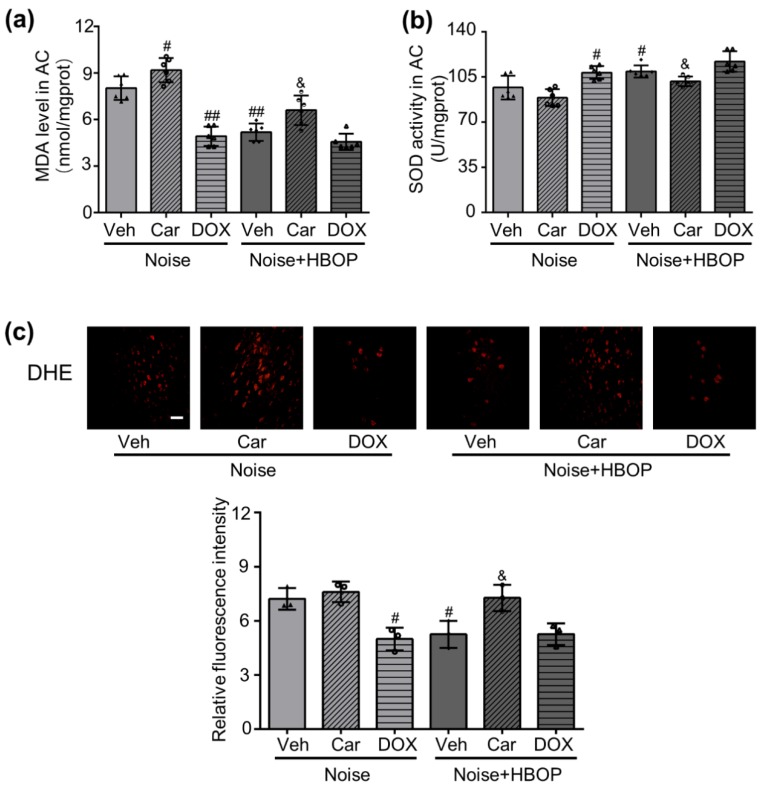
Summarized data showing malonaldehyde (MDA) content (**a**) and superoxide dismutase (SOD) activity (**b**), original micrographs and analyzed data (**c**) on superoxide-sensitive dihydroethidium (DHE) fluorescence in auditory cortex of mice from Noise or HBOP group treated with Veh, Car, or DOX detected by colorimetric (**a**,**b**) or fluorescence staining (**c**) assay. Magnification ×400, scale bar = 50 μm. Data are shown as means ± SD, *n* = 6 in colorimetric assay, *n* = 3 in fluorescence staining, ^##^*P* < 0.01, ^#^*P* < 0.05 vs Noise, ^&^*P* < 0.05 vs. HBOP. All individual data points were shown in the figure using different shapes, ▲ for Noise group, ◯ for Noise+Car group, ∆ for Noise+DOX group, ⬥ for Noise+HBOP group, ◓ for Noise+HBOP+Car group, ◭ for Noise+HBOP+DOX group.

To identify the downstream event of Cer, oxidative stress and antioxidative potency of AC were measured. As shown in Figure 5a,c, HBOP reduced both the MDA content and superoxide production in noise-exposed mice, and this effect was reversed significantly by Car treatment (*P* < 0.01, *P* < 0.05, *P* < 0.05). Car also enhanced the MDA content in Noise group (Figure 5a, *P* < 0.05). On the contrary, DOX reduced MDA and superoxide production in Noise group, but did not change those in Noise with HBOP group (Figure 5a,c, *P* < 0.01, *P* < 0.05). As for SOD activity, HBOP enhanced it in Noise group, which was restored by Car treatment (Figure 5b, *P* < 0.05, *P* < 0.05). However, the Car did not change the SOD activity in noise group. DOX increased SOD activity in noise group (Figure 5b, *P* < 0.05), but did not adjust it in Noise with HBOP group.

### 2.6. Vc Neutralized Oxidative Stress, Restoring the Hearing Loss and Neuronal Damage in AC of Noise Exposed Mice

**Figure 6:**
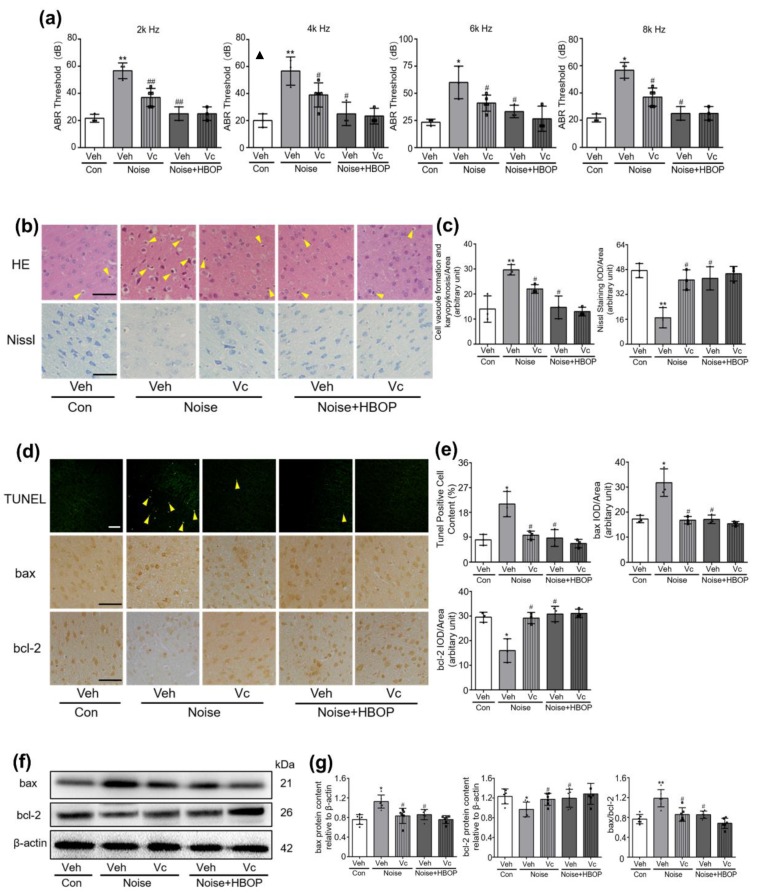
ABR threshold to sound with frequency of 2k, 4k, 6k, or 8k Hz (**a**), original images (**b**), and summarized data (**c**) of HE or Nissl staining in AC of mice in Con + Veh, Noise + Veh, Noise + Vc, Noise + HBOP + Veh, or Noise + HBOP + Vc group. The arrows indicate the vacuole formation and karyopyknosis in the neuron. Original images (**d**,**f**) and summarized data (**e**,**g**) showing neuronal apoptosis, bax, and bcl-2 in AC of mice in Con, Noise, and Noise + HBOP group treated with Veh or Vc detected by TUNEL (**d**), IHC (**d**), or western blotting (**f**) assay. The arrows indicate apoptotic neurons, magnification ×200, in HE, Nissl or IHC, magnification ×400, scale bar = 50 μm. Data are shown as means ± SD, *n* = 3 in HE, Nissl, IHC or TUNEL, *n* = 6 in western blotting assay, **P < 0.01, *P < 0.05 vs Con, ^##^*P* < 0.01, ^#^*P* < 0.05 vs Noise. All individual data points were shown in the figure using different shapes, • for Con group, ▲ for Noise group, ⬥ for Noise+HBOP group, ⬒ for Noise+Vc group, ◧ for Noise+HBOP+Vc group.

Vc reduced the MDA and superoxide production, enhancing the SOD activity in AC of Noise group (*P* < 0.05), but not the Noise with HBOP group (Supplementary Figure S5). Accordingly, in noise-exposed mice, Vc treatment reduced the ABR threshold on all the frequencies tested, while ineffective in Noise with HBOP group (Figure 6a, *P* < 0.01, *P* < 0.05). With Vc administration, neuronal morphology was improved significantly, and Nissl test detected more living neurons in AC of noise-exposed mice (Figure 6b,c, *P* < 0.05). Similarly, Vc showed no significant impact on both neuronal morphology and living cell amount in Noise with HBOP group (Figure 6b,c).Moreover, after Vc gavage, the percentage of apoptotic neurons was decreased significantly (Figure 6d,e, *P* < 0.01), which was accompanied with lower protein expression of bax and higher expression of bcl-2 in AC of noise-exposed mice (Figure 6d–g, *P* < 0.01, *P* < 0.05).

### 2.7. Vc Normalized the Cer Generation in AC of Noise Exposed Mice

**Figure 7:**
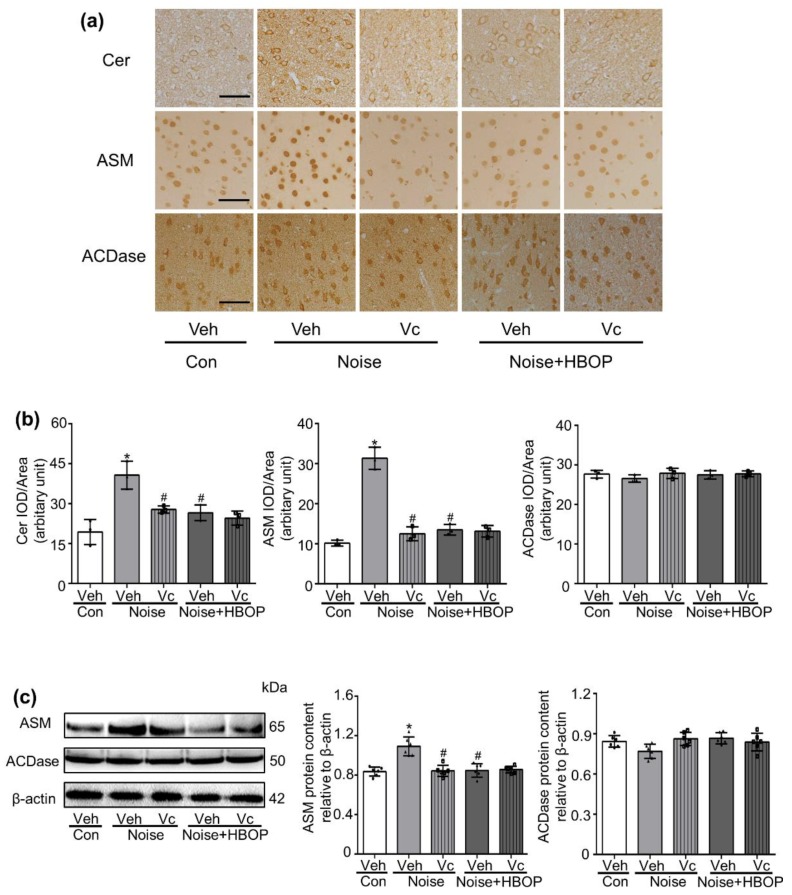
Representative images and summarized data showing content of Cer (**a**,**b**), ASM (**a**–**c**) or ACDase (**a**–**c**) in AC of mice from Con + Veh, Noise + Veh, Noise + Vc, Noise + HBOP + Veh, or Noise + HBOP + Vc group detected by IHC (**a**) or western blot (**c**) assay. Magnification ×400, scale bar = 50 μm. Data are shown as means ± SD, *n* = 3 in IHC, *n* = 6 in western blot, *P < 0.05 vs. Con, ^#^*P* < 0.05 vs Noise. All individual data points were shown in the figure using different shapes, • for Con group, ▲ for Noise group, ⬥ for Noise+HBOP group, ⬒ for Noise+Vc group, ◧ for Noise+HBOP+Vc group.

As shown in Figure 7a,b, Cer generation enhanced by noise exposure was reduced significantly by Vc (*P* < 0.05). In the meantime, both IHC and Western blot analysis showed a lower ASM protein expression in Noise with Vc group than Noise group (Figure 7a–c, *P* < 0.05). ACDase responded to neither the HBOP nor Vc treatment. In Noise with HBOP group, Vc administration showed no influence on all the items including Cer abundance, ASM, and ACDase expression.

## 3. Discussion

In this study, we demonstrated the HBOT, especially when imposed prior to noise exposure, restored the impaired auditory sensitivity, neuronal morphology, and apoptosis in AC. Further to this, the mutual regulation between Cer generation and oxidative stress-mediated the advantages of HBOT.

Noise engenders not only hearing loss but also tinnitus, hyperacusis, and poor speech intelligibility, which would be greatly assigned to the neuronal disorder in central auditory pathway [1,18]. Evidence from both human and animal studies indicate that noise reduces cell density, brings on prominent disorder or apoptosis within central auditory pathway and retards development of primary auditory cortex [1,2,19]. Our former research revealed the broad band (20-20k Hz) noise with average intensity of 95 dB SPL caused hearing loss over frequencies from 4k to 32k Hz, accompanied by degenerative remodeling of neurons in AC, including swollen cell, widespread vacuole formation, and nuclei deviation from the center [3].

Protection on auditory cortex should be critical in the preservation of hearing and neuronal vitality. Medications such as anti-oxidants, calcineurin inhibitors, diuretics, glucocorticoids, c-Jun N-terminal kinase (JNK) inhibitors, magnesium, N-methyl-D-aspartic acid receptor (NMDA) antagonists and nitric oxide synthase (NOS) inhibitors were shown more or less efficient in either laboratory or clinic studies [20]. Nevertheless, hearing loss due to the occupational noise exposure in workplaces is still a crucial public health issue [21]. HBOT acts as a non-invasive therapeutic procedure where patients breathe 100% oxygen under atmospheric pressure higher than 1 ata, which is beneficial to multiple neuronal maladies [22,23,24]. To date, based on the recommendation of Hyperbaric Oxygen Therapy Committee of the Undersea and Hyperbaric Medical Society, U.S. Food and Drug Administration (FDA) has approved of applying HBOT in 14 clinical conditions including air or gas embolism, carbon monoxide poisoning, crush injury and delayed radiation injury [25]. Although sudden deafness does not show up in the list, HBOT has been widely accepted as a primary or salvage therapy since 1970s [26]. The underlying mechanism might relate to the elevated oxygen tension in perilymphatic fluid, which is 4.5 times higher under 2 ata HBOT than breathing air under 1 ata [27].

In the present work, 7d noise exposure raised the ABR threshold of the sound on both low and high frequencies, which indicates a fallen hearing capacity. Withdrawal from the noise for seven days did not recover the situation. Among the three types of HBOTs, HBOP, but not HBOD or HBOA improved the hearing substantially, suggesting the oxygen reservation before noise exposure might be more powerful in protecting auditory sensitivity than oxygen replenishment, which as far as we have known, has never been reported before. Next, we found the disarrangement of morphology and configuration of neurons in AC was also just responsive to HBOP, but not HBOD or HBOA (Figure 1b,c). This result agrees with reports showing dependence of hearing capacities on rational morphology of the auditory system [28]. Also, it is consistent with the study proving HBO preconditioning as an effective and feasible approach in preventing, alleviating, and improving postoperative cognitive dysfunction [29]. However, in the Nissl staining and apoptosis detection, AC was improved by all the three types of HBOT (Figure 1b,c) suggesting the viability of individual neuron might be easier to recover than the organization of multiple neurons. This result underscores the contribution of neuronal connection and configuration to cortex functionality of AC.

As for the mechanism behind the curative action of HBOT, enhancement of oxygen tension in local tissue should be the core one, but candidates far beyond this had been proposed. Vascular endothelial growth factor, hypoxia-inducible factor, stem/progenitor cells, heme oxygenase-1, or heat shock protein might play a role [5]. Moreover, HBOT maintains cell viability in the ischemic border zone, restrains mitochondrial dysfunction, recovers the metabolic change in penumbra, and blocks inflammatory cascades in acute stroke [30]. Sphingolipids (SLs) serves as an essential constituent of membrane in eukaryotic cells, which, being a crucial signaling molecule, controls key cellular processes as apoptosis, autophagy, proliferation, differentiation, and migration inside cell [31]. Cer lies at the very center of SLs metabolism which retains a pro-apoptotic characteristic. The abundance of Cer relies mostly on the balance between ASM and ACDase, enzymes responsible for its synthesis and degradation [11]. Our former research demonstrated that the hearing loss and neuronal damage upon noise exposure were attributable to ASM over-activation and Cer accumulation in AC [3]. In this work, we found HBOT reduced ASM protein expression without affecting that of ACDase, and caused a lower Cer content in AC of noise-exposed mice, suggesting the compromised generation but not the extra degradation motivates the Cer accrual. Furthermore, when Car was adopted to enhance the Cer accumulation, the benefits of HBOT on hearing loss or neuronal damage was discounted. On the contrary, ASM inhibition by DOX minimized the neuronal disorder in noise-exposed mice just like the HBOT did. All these evidence uphold the possibility that the ASM/ACDase/Cer pathway was responsible for the action of HBOT on mitigating the hearing loss and AC neuronal damnification. We did not explore how the HBOT perturbed ASM/ACDase/Cer but inferred the high oxygen tension acted as a stimulus to intrude on natural behavior of lysosomes [32] where the ASM is mainly perched, which might evoke hydrolysis of the protein by proteolytic enzymes.

Cer fortifies the oxidative stress by promoting aggregation of NADPH oxidase subunits [9] or the mitochondria imbalance [33]. Usually, HBOT has been taken for granted to enhance the oxidative stress in tissue. However, there are quite a few controversial views. For example, HBOT neutralized anti-oxidant enzymes exacerbating oxidative stress in rat with diabetes mellitus [34]. It promoted both oxidant and anti-oxidant stress in rat lung [35], but did not influence oxidative stress in children with autism [36] or the mice with pain [17]. It even exerted positive effects on liver regeneration by decreasing MDA or facilitating anti-oxidant potency [37]. Furthermore, it minified oxidative markers in brain trauma and alleviated the cerebral toxicity [38]. In the present work, HBOT reduced the superoxide and MDA formation, showing no detriment to anti-oxidants in noised exposed mice, which was reversed by Cer promotion. Meanwhile, ASM inhibition behaved similarly as HBOT on reducing oxidative stress, indicating the ASM/ACDase/Cer pathway accounted for the effect of HBOT on oxidative stress. Next, Vc, a widely used over-the-counter anti-oxidant, reduced the neuronal damage and improved hearing capability in noise but not HBOT group, which along with other evidence strengthens the viewpoint that the oxidative stress lies downstream of Cer accounting for the benefits of HBOT on noise provoked hearing loss and neuronal disorder.

In multiple studies, oxidative stress and Cer responds simultaneously to chemical or physical stimulators such as inflammatory cytokines and mechanical stresses [39], implying a mutual modulation between the two critical entities. In the present work, with the bluntness of oxidative stress, ASM expression and Cer production dropped significantly, signifying a reciprocal regulation of oxidative stress on Cer. Our result was in line with reports on aging brain and Alzheimer’s disease [40] which revealed the involvement of oxidative stress in maladjusted Cer metabolism. Likewise, in the process of cellular apoptosis upon H_2_O_2_ incubation or hypoxia, Cer generation has turned out to be a committed step [41]. Apart from a well-known anti-oxidant, Vc holds visible benefits in myelin formation, neuronal maturation, differentiation, catecholamine synthesis, neurotransmission modulation, embryonic and postnatal neuronal development [42]. Our findings in such a manner put forward new machinery for the function of Vc and suggest the people suffering from noise pollution might take Vc regularly as a countermeasure to preserve hearing and auditory neuronal wellness.

In general, our work demonstrated 7 d broad band noise exposure induced hearing loss and neuronal damage which could be recovered by HBOT, especially HBOP. The therapeutic effect of HBOT was attributed to lowered Cer production, blunted oxidative stress, and the mutual regulation between them in AC.

## 4. Materials and Methods

### 4.1. Materials

Carmofur (Car) (Cat# S1289) was purchased from Selleck (Houston, TX, USA). Doxepin (DOX) and Vitamin C (Vc) were obtained from XinYi Pharmaceutical Co. Ltd. (Shanghai, China) and LiJun Pharmaceutical Co. Ltd. (Xi’an, Shaanxi, China), respectively. Rabbit polyclonal antibody against ACDase (Cat# 11274-1-AP,) and Bcl-2 Associated X Protein (bax) (Cat# 50599-2-Ig) were purchased from Proteintech Group (Rosemont, IL, USA). Rabbit monoclonal antibody specific for B cell lymphoma/lewkmia-2 (bcl-2) (Cat# ab182858) and mouse monoclonal antibody against ASM (Cat# ab74281) were purchased from Abcam (Cambridge, MA, USA). Mouse monoclonal antibody against Cer (Cat# ALX-804-196) was obtained from Enzo Life Sciences (Farmingdale, NY, USA). The TUNEL kit (Cat# C1088) was purchased from Beyotime (Shanghai, China). Malondialdehyde (MDA) testing kit (Cat# A003-1) and superoxide dismutase (SOD) activity testing kit (Cat# A001-1) were obtained from Nanjing Jiancheng Bioengineering Institute (Nanjing, Jiangsu, China). The SP Histostain-Plus Kits (Cat# SP-9001, SP-9002, ZLI-9018) were purchased from ZSGB-Bio (Beijing, China).

### 4.2. Animals

Chinese Kun Ming (KM) mice (male, weighing 20–25 g) were obtained from the Laboratory Animal Center of the Fourth Military Medical University (FMMU) of China. All animals were detected without hearing deficits. Mice were housed 6 in a cage, maintained within a 12 h:12 h light/dark cycle at 23 ± 1 °C with standard lab chow and water available ad libitum. In this study, all procedures and protocols were conducted in agreement with the National Institutes of Health guidelines for the care and use of laboratory animals (NIH Publications No. 8023, revised 1978). Meanwhile, all the procedures in the present study were reviewed and approved by the Bioethical Committee for Animal Care at the FMMU (No. 20160904).

### 4.3. Animal Experimental Groups and Experimental Design

In the first part of experiments, after 3 days of accommodation, 42 mice were randomly divided into 7 groups: ① control (Con) group, ② HBOT without noise exposure (Con + HBO) group, ③ Noise group, ④ no intervention after noise exposure (Noise untreated) group, ⑤ HBOT before noise exposure (Noise + HBOP) group, ⑥ HBOT during noise exposure (Noise + HBOD) group and ⑦ HBOT after noise exposure (Noise + HBOA) group. The duration of HBOT was 7 days. Group ② received HBO (2.5 ata, 60 min) therapy alone. Group ③, ④, ⑤, ⑥, ⑦ were exposed to broad band noise (20–20k Hz, 95 dB, 3 h/day) for 7 days. Group ⑤ received HBO for 7 days before noise exposure while group ⑥ during and ⑦ after. After noise exposure, mice in group ③ were executed immediately while group ④ were raised for 7 days without extra treatments. All procedures were conducted following the timeline in Supplementary Figure S1a.

In the second part of experiments, 36 mice with earmarks were divided into 6 groups randomly, 6 in each group. All the mice were exposed to noise while 3 groups were undergoing HBOT before noise exposure. At the same time, 24 mice were treated by Car and DOX respectively. Car (22.4 mg kg^−1^) [43,44] was injected intraperitoneally every other day and DOX (5 mg kg^−1^) [3] was given intragastrically daily 3 days before till the end of the noise exposure. The other mice were given vehicle (Veh) at the same time. Procedures were conducted following the timeline in Supplementary Figure S1b.

In the third part of experiments, 30 mice were randomly divided into 5 groups, that is Con + Veh, Noise + Veh, Noise + Vc, Noise + HBOP + Veh and Noise + HBOP + Vc. Except the Con + Veh, the other 4 groups were exposed to noise for 7 days, treated by Vc or HBO or both. Vc (200 mgkg^−1^ day^−1^) [45,46] was given intragastrically 7 days before till the end of the noise exposure. All steps were performed following the timeline in Supplementary Figure S1c.

### 4.4. Noise Exposure

The noise we used has been described in a recent publication [3]. In brief, mice were exposed to a recorded broadband noise (3 h, 20–20k Hz, 95 dB on average) in a soundproof room. A CD player (Hualu Technology Co. Ltd., Dalian, China) was connected to a loudspeaker (Aidefa Technology Co. Ltd., Beijing, China) placed 30 cm away from the laboratory cages in which the mice were raised unrestrainedly. A sound meter (Hengsheng Electronic Co. Ltd., Jiaxing, China) was adopted to measure the intensity of the noise played through the loudspeaker to ensure the conformance of stimulation.

### 4.5. Hyperbaric Oxygen Therapy

The mice in cages were placed into an animal hyperbaric oxygen chamber (Capacity: 45 cm × 45 cm × 75 cm) (Yangyuan Manufacturing, DWC450-1150, Shanghai, China). After several minutes of accommodation, the pressure inside the chamber was elevated to 2.5 ata with compressing 100% oxygen in 10 min. Then the pressure was kept at the level for 60 min, which was followed by 10 min of decompression to 1 ata. During the treatment, the mice breathed spontaneously without getting anesthetization. Simultaneously, they were closely monitored to maintain good condition.

### 4.6. Auditory Brainstem Response (ABR) Assay

To determine the hearing potency, threshold of ABR was examined. Mice were anesthetized by intraperitoneal injection of pentobarbital sodium (50 mg/kg) and then were fixed on a heating pad maintained at 37 °C as described previously [3] to minimize post-procedural suffering. The active, reference, and ground electrodes were placed subcutaneously in the vertex, auricle of the tested ears, and tail respectively. The test was conducted in a sound-attenuating room using an ABR Workstation (Otometrics, Taastrup, Denmark) where the response to sounds with frequency of 2k, 4k, 6k, or 8k Hz was recorded. The sound intensity was first set at 80 dB and then decreased gradually by 10 dB. When the threshold was approached, the decreasing amplitude was changed into 5 dB. The lowest intensity on which the ABR could be detected was regarded as ABR threshold. The representative ABR waveform was shown in Supplementary Figure S2.

### 4.7. Tissue Preparation

After the ABR test, the left ventricle of KM mice was perfused with normal saline until the blood drained away after the chest was opened. Then the brain was rapidly removed and kept on the ice. For staining tests, brains were kept in 4% paraformaldehyde (PFA) solution overnight and then embedded in paraffin or optimal cutting temperature compound (O.C.T.). Coronal sections containing AC (shown in Supplementary Figure S3) were prepared on a microtome, which was for hematoxylin-eosin (HE), Nissl staining, immunohistochemistry (IHC), dihydroethidium (DHE), or transferase-mediated dUTP nick end labeling (TUNEL) examination. Other brains were carefully dissected, and the auditory cortexes were kept at −80 °C for other tests.

### 4.8. Hematoxylin Eosin (HE) Staining

The 5 μm brain sections were deparaffinized, hydrated through gradient alcohol and then washed in distilled water. After staining with hematoxylin for 2 min, the sections were rinsed in running tap water for 1 min. Next, the differentiation with acid alcohol, 20 s staining with eosin, dehydration and mounting with neutral resins were conducted in order. Finally, the photographs were taken under a light microscope (DP71, Olympus, Japan) and viewed using Image J 1.4.3.67 software (Broken Symmetry Software, Scottsdale, AZ, USA).

### 4.9. Nissl Staining

After deparaffinization and hydration in gradient alcohol, the brain sections were stained by 1% toluidine blue dye for 10 min, and then were washed in distilled water for 1 min. Next, the sections were dehydrated in gradient alcohol and mounted with neutral resins. Living neurons with blue staining was observed under a light microscope (DP71, Olympus, Japan) and analyzed with the Image J 1.4.3.67 (Broken Symmetry Software, Scottsdale, AZ, USA).

### 4.10. Transferase-Mediated dUTP Nick end Labeling (TUNEL) Staining

The TUNEL kit was applied to detect apoptotic cells following the instructions. The sections were incubated in dark moist boxes for 60 min at 37 °C with Terminal Deoxynucleotidyl Transferase (TdT), labeling 3′-OH DNA termini with modified nucleotides, and this reaction was stopped by immersing the slide in wash buffer for 10 min. The TUNEL-positive cells showing green fluorescence were photographed with a fluorescent microscope (DP71, Olympus, Japan), which were counted manually.

### 4.11. Immunohistochemistry (IHC) Assay

IHC assay was performed following the instructions of the SP Histostain-Plus Kits. After deparaffinization and hydration, the sections were first treated in 3% H_2_O_2_ for 10 min and then were incubated with reagent A for 15 min at room temperature. Subsequently, antibodies against bax, bcl-2, Cer, ASM, or Cer was dripped on the sections for incubation for 60 min at 37 °C. After three times of PBS wash, the sections were incubated with reagent B and then C for 15 min each at 37 °C. Finally, the sections were treated by DAB for 10 min, dehydrated and mounted with neutral resins. The photographs were taken under a light microscope (DP71, Olympus, Japan) and analyzed with Image J 1.4.3.67 (Broken Symmetry Software, Scottsdale, AZ, USA).

### 4.12. Western Blot Analysis

The brain samples were minced and homogenized on ice in protein extraction reagent (Cat# 78501, Thermo, Rockford, IL, USA) with the aid of tissue grinders. Then the samples were centrifuged at 12,000 rpm for 10 min at 4 °C with the supernatants collected afterward. Protein concentrations were determined with a Pierce^TM^ Bicinchoninic Acid Assay Kit (Cat# 23228, Thermo, Rockford, IL, USA). 30 μg proteins from different groups were loaded to 4–12% Bis-Tris PAGE gels under denaturing conditions within the NuPAGE Bis-Tris Pre-cast Gel System (Invitrogen Life Technologies, Carlsbad, CA, USA). After electrophoresis, the proteins were transferred to polyvinylidene difluoride (PVDF) membranes (Millipore, Billerica, MA, USA) in an XCell Blot Module transfer system (Invitrogen Life Technologies, Carlsbad, CA, USA). Then, 5 % BSA in TBS containing 0.1 % (*w*/*v*) Tween 20 (TBS-T) was used to blocking the membranes. Next, we incubated the membranes with primary antibodies, namely rabbit polyclonal antibody against ACDase, β-actin, bax, bcl-2, and ASM in blocking buffer at 4 °C overnight. After six times of washing with TBS-T and 90 min incubation at room temperature with secondary antibodies as horseradish peroxidase (HRP) conjugated goat anti-rabbit or anti-mouse IgG (1:10,000, Proteintech, Rosemont, IL, USA), the membranes underwent exposure of the bands by detection reagents (Cat# WBKLS0100, Millipore, Billerica, MA, USA) in a Gel Image Analyzing System (Tanon Science and Technology, Shanghai, China) which was followed by 6 time washing in TBS-T. Densitometry analysis was performed with Image J 1.4.3.67 (Broken Symmetry Software, Scottsdale, AZ, USA).

### 4.13. Detection of Lipid Peroxidation, Superoxide Dismutase (SOD) Activity

Both Malondialdehyde (MDA) level, one of the indexes of lipid peroxidation, and superoxide dismutase (SOD) activity were examined using the commercial testing kit (Cat# A003-1, Nanjing Jiancheng Bioengineering Institute, Nanjing, China) according to manufacturer’s instruction. The chromaticity was monitored with a microplate reader (Thermo Fisher, Waltham, MA, USA) at 532 nm or 550 nm.

### 4.14. Detection of Superoxide Production

The 10 μm frozen coronal brain sections were incubated with 10 μM dihydroethidium (DHE) for 20 min at 37 °C, and after wash, they were viewed under fluorescence microscopy (Olympus, Japan). The intensity of DHE fluorescence was analyzed by Image J 1.4.3.67 (Broken Symmetry Software, Scottsdale, AZ, USA).

### 4.15. Statistical Analysis

All data are presented as means ± SD. The exact number of mice for each experiment is given in the figure legends. A test to identify normality and outliers was not performed. Comparisons of two groups were done with two-tailed Student’s t tests and comparisons of multiple groups were done with ANOVA by using the software Statistical Program for Social Science (SPSS) 16.0 (IBM, Chicago, IL, USA). GraphPad Prism version 7.0 (GraphPad Software Inc, San Diego, CA, USA) was used for graphics. A value of *P* < 0.05 was defined as being statistically significant.

## Supplementary Materials

Supplementary materials can be found at https://www.mdpi.com/1422-0067/20/19/4675/s1.

## Abbreviations

A1	Primary auditory cortex
ABR	Auditory brainstem response
AC	auditory cortex
ACDase	acid ceramidase
ASM	acid sphingomyelinase
bcl-2	B cell lymphoma 2
bax	bcl-2 associated X protein
Car	Carmofur
Cer	Ceramide
DOX	doxepin hydrochloride
FDA	U.S. Food and Drug Administration
HBOT	hyperbaric oxygen therapy
HBOA	hyperbaric oxygen therapy after noise exposure
HBOD	hyperbaric oxygen therapy during noise exposure
HBOP	hyperbaric oxygen therapy before noise exposure
HE	hematoxylin-eosin
IHC	immunohistochemistry
JNK	c-Jun N-terminal kinase
MDA	malondialdehyde
MGB	medial geniculate body
NMDA	N-methyl-D-aspartic acid receptor
NOS	nitric oxide synthase
SLs	sphingolipids
SOD	superoxide dismutase
TUNEL	transferase-mediated dUTP nick end labeling
Vc	Vitamin C
Veh	Vehicle
