# Supplementary figures and images for: Hyperbaric Oxygen Treatment Ameliorates Hearing Loss and Auditory Cortex Injury in Noise Exposed Mice by Repressing Local Ceramide Accumulation
Source: Int J Mol Sci. 2019 Sep 20;20(19):4675. doi: 10.3390/ijms20194675 (PMC6801451; doi:10.3390/ijms20194675)

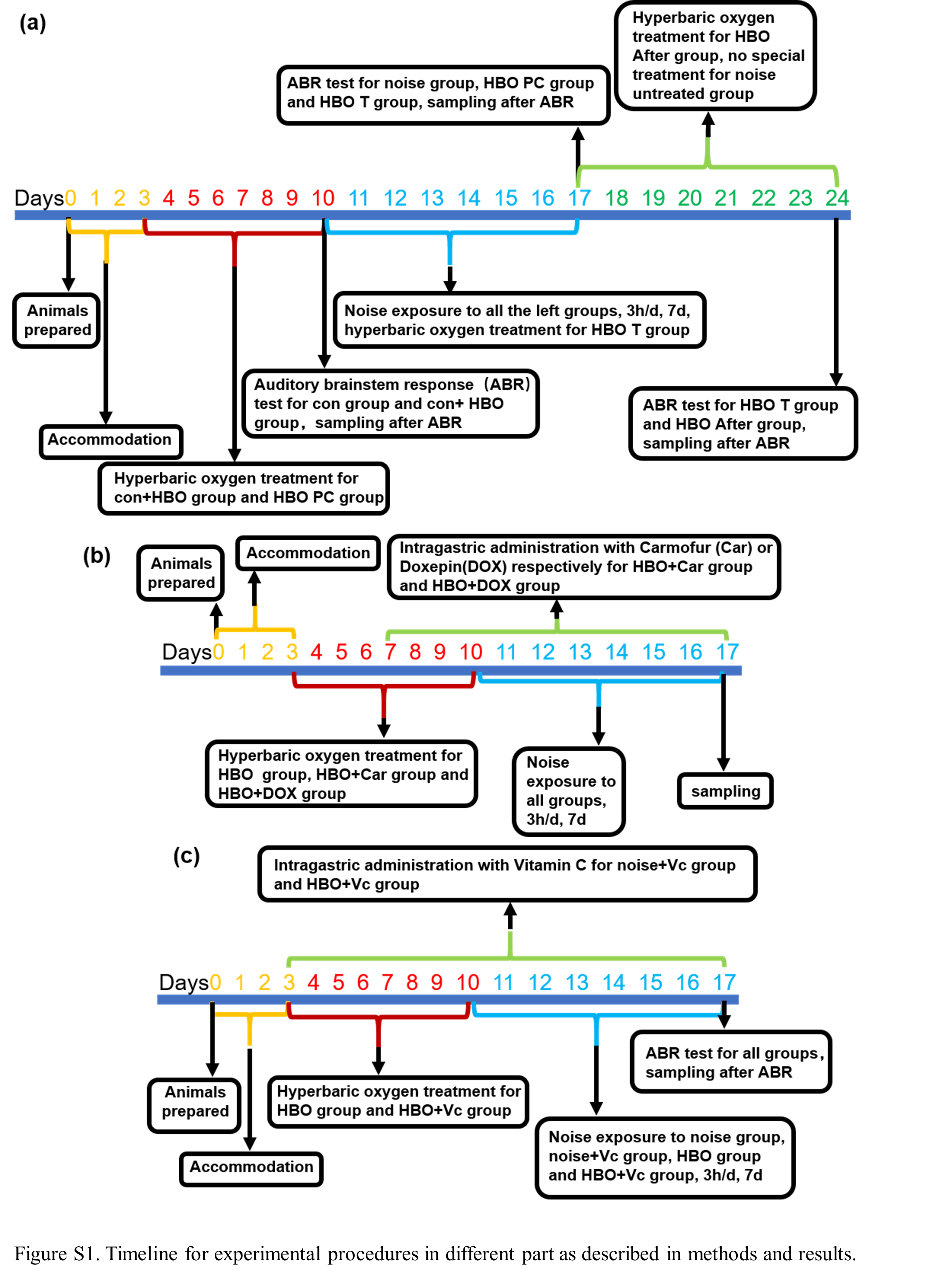

Supplement: Supplementary file 1 [file ijms-20-04675-s001.zip › ijms-563207 supplementary/Supplementary Files/Figure S1.TIF]

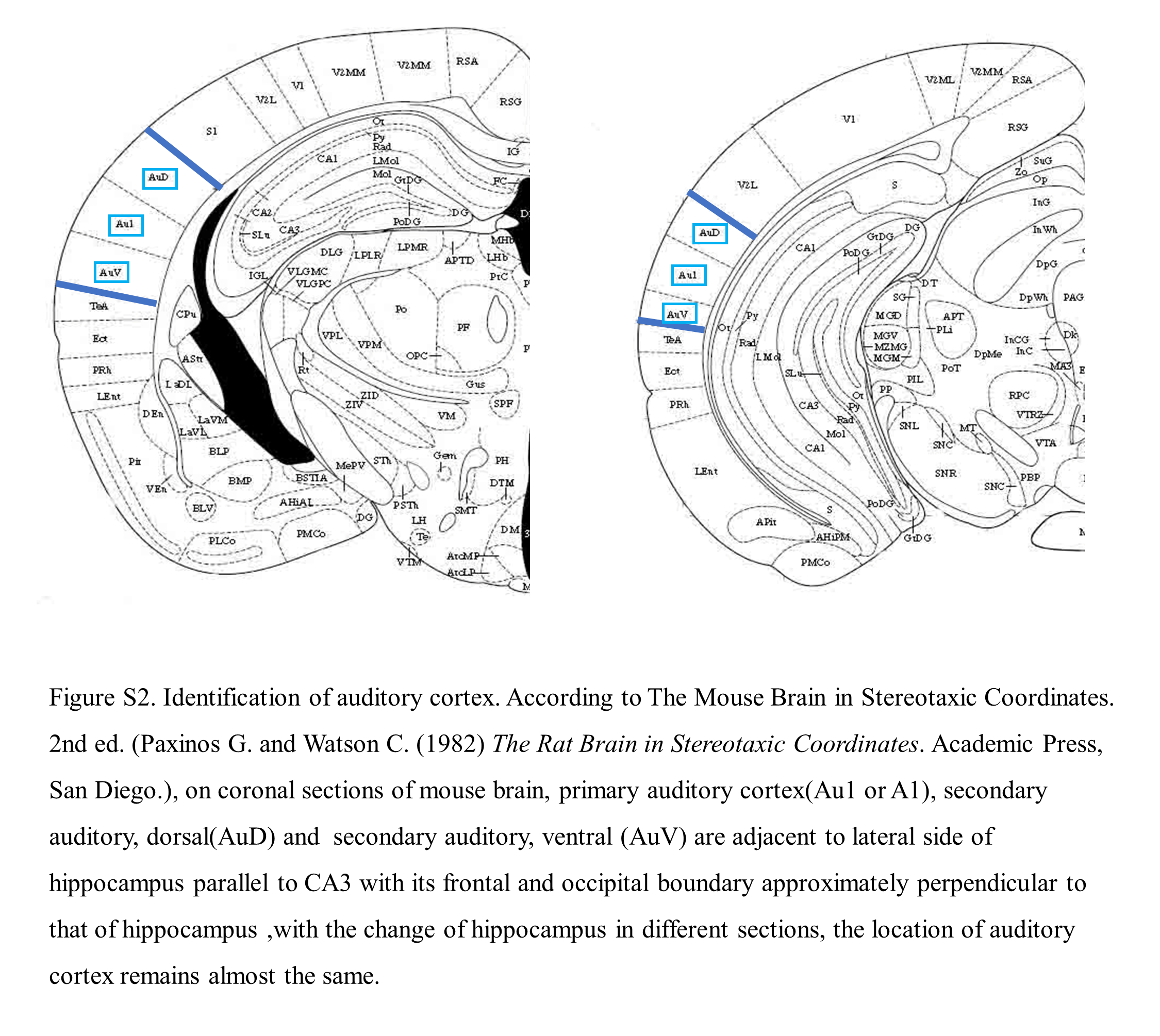

Supplement: Supplementary file 1 [file ijms-20-04675-s001.zip › ijms-563207 supplementary/Supplementary Files/Figure S2.TIF]

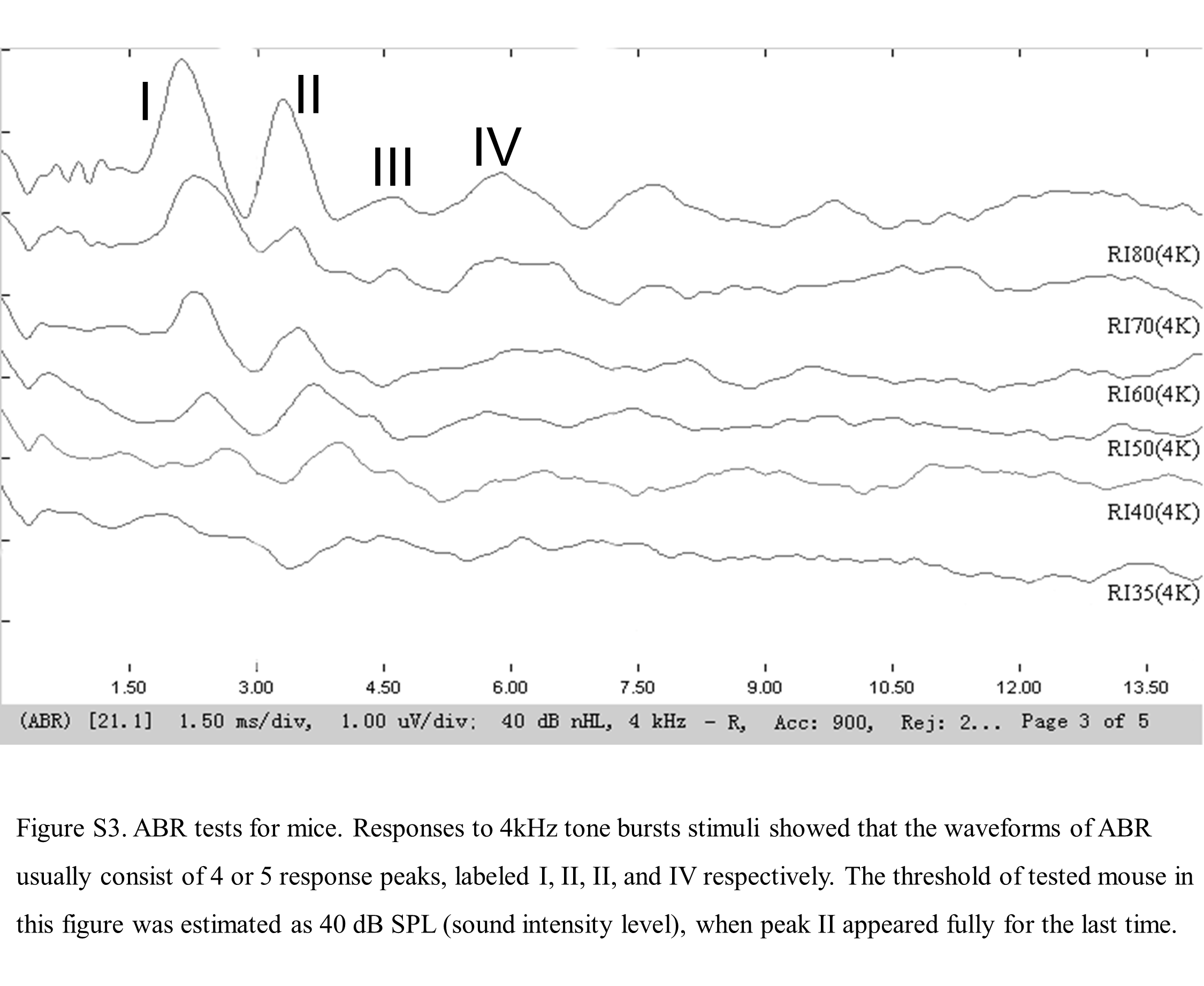

Supplement: Supplementary file 1 [file ijms-20-04675-s001.zip › ijms-563207 supplementary/Supplementary Files/Figure S3.TIF]

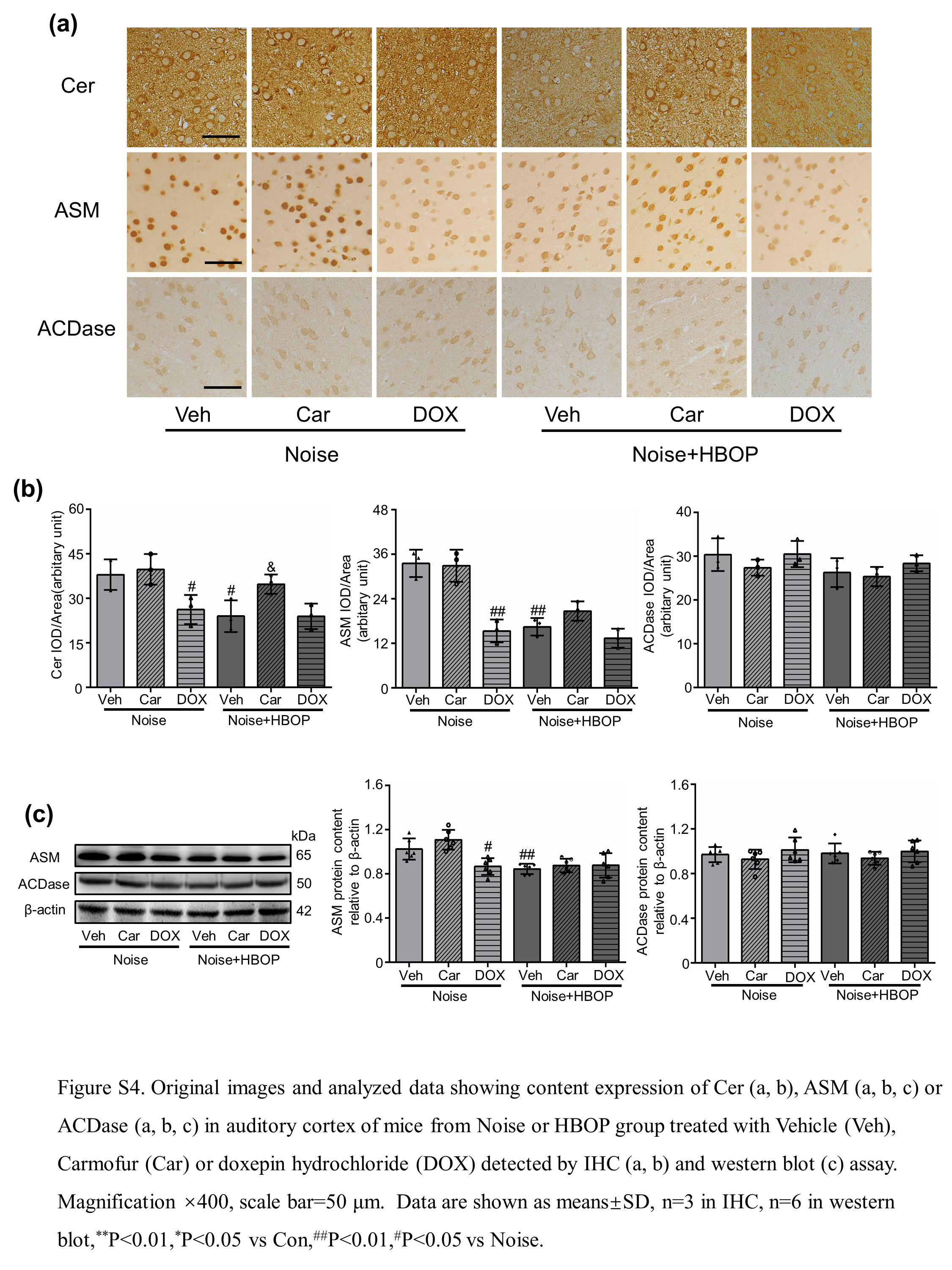

Supplement: Supplementary file 1 [file ijms-20-04675-s001.zip › ijms-563207 supplementary/Supplementary Files/Figure S4.TIF]

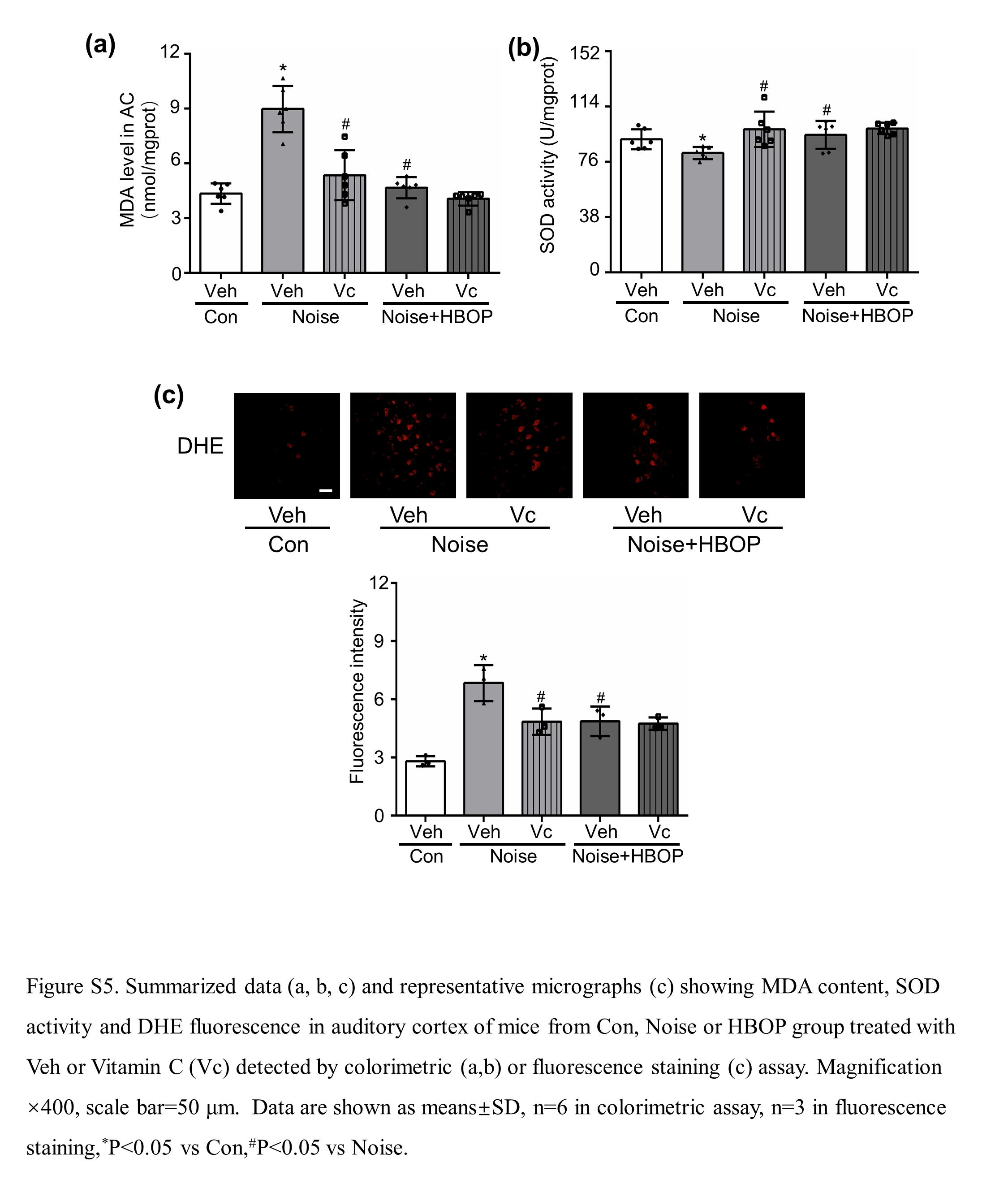

Supplement: Supplementary file 1 [file ijms-20-04675-s001.zip › ijms-563207 supplementary/Supplementary Files/Figure S5.TIF]
